# Digitale Technologien in der primären Knieendoprothetik – Nutzungsdichte im deutschsprachigen Raum

**DOI:** 10.1007/s00132-024-04575-7

**Published:** 2024-11-01

**Authors:** Florian Pohlig, Roland Becker, Max Ettinger, Tilman Calliess, Florian Hinterwimmer, Carsten O. Tibesku, Christoph Schnurr, Heiko Graichen, Peter Savov, Stefano Pagano, Ralf Bieger, Hans Gollwitzer

**Affiliations:** 1 Klinik für Orthopädie und Sportorthopädie, Klinikum rechts der Isar, Technische Universität München, Ismaninger Str. 22, 81675 München, Deutschland; 2 Zentrum für Orthopädie und Unfallchirurgie, Endoprothesenzentrum West-Brandenburg, Universitätsklinikum Brandenburg an der Havel, Hochstr. 29, 14770 Brandenburg an der Havel, Deutschland; 3 Klinik für Orthopädie und Unfallchirurgie, Pius Hospital Oldenburg, Universitätsmedizin Oldenburg, Georgstr. 12, 26121 Oldenburg, Deutschland; 4 articon Spezialpraxis für Gelenkchirurgie, Berner Prothetikzentrum, Schänzlistrasse 39, 3013 Bern, Schweiz; 5 KniePraxis Prof. Dr. Tibesku, Bahnhofplatz 1, 94315 Straubing, Deutschland; 6 St. Vinzenz Krankenhaus Düsseldorf, Schloßstr. 85, 40477 Düsseldorf, Deutschland; 7 Privatklinik Siloah, Orthopädie und Traumatologie, Worbstr. 324, 3073 Gümlingen, Schweiz; 8 Orthopädische Klinik, Universität Regensburg, Asklepios Klinikum Bad Abbach, Kaiser-Karl-V.-Allee 3, 93077 Bad Abbach, Deutschland; 9 Schön Klinik München Harlaching, Harlachinger Str. 51, 81547 München, Deutschland; 10 ECOM – Praxis für Orthopädie, Sportmedizin und Unfallchirurgie, Arabellastraße 17, 81925 München, Deutschland

**Keywords:** Augmented Reality, Computerassistierte Chirurgie, Robotik, Chirurgische Navigationssysteme, Totaler Kniegelenkersatz, Augmented reality, Computer-assisted surgery, Robotics, Surgical navigation systems, Total knee replacement

## Abstract

**Hintergrund:**

Digitale Assistenzsysteme werden weltweit zunehmend in der primären Knieendoprothetik eingesetzt. Ziel war es, die Nutzungsdichte digitaler Hilfsmittel, die bevorzugten Alignmentstrategien sowie die Hindernisse und Vorteile der Implementierung dieser Technologien im deutschsprachigen Raum zu analysieren.

**Material und Methoden:**

Es wurde eine Online-Umfrage mit 57 Fragen zu digitalen Tools in der primären Knieendoprothetik sowie deren Nutzung unter den Mitgliedern der Arbeitsgemeinschaft Endoprothetik (AE) durchgeführt. Folgende Technologien und deren Nutzung wurden abgefragt: Navigation, Robotik, patientenspezifische Instrumente, Individualimplantate sowie die Nutzung von Augmented Reality.

**Ergebnisse:**

Die Umfrage ergab, dass 18 % der Kliniken Navigations- und 17 % Robotiksysteme in der primären Knieendoprothetik einsetzen. Die Hauptgründe für die Nichtnutzung dieser Technologien waren die hohen Anschaffungs- und laufenden Kosten sowie der zusätzliche perioperative Zeitaufwand. Patientenspezifische Instrumente und Individualimplantate spielen aktuell nur eine untergeordnete Rolle in Deutschland. Patientenindividuelle Alignmentstrategien wie kinematisches (Navigation: 35 %; Robotik: 44 %) und funktionelles Alignment (Navigation: 15 %; Robotik: 35 %) werden bei Einsatz digitaler Assistenzsysteme bevorzugt. Dies stellt einen deutlichen Unterschied zur konventionellen Operationsmethodik dar, bei der die klassische mechanische Ausrichtung der Prothese dominiert (79 %).

**Diskussion:**

Die Ergebnisse zeigen eine vergleichsweise hohe Nutzungsdichte digitaler Tools, die von den Operateuren genutzt werden, um personalisierte Alignmentstrategien in der primären Knieendoprothetik im deutschsprachigen Raum umzusetzen. Dies konnte insbesondere für Zentren mit hohem Operationsvolumen gezeigt werden. Gegen die Nutzung wurden vorrangig ökonomische Gründe aufgeführt. Zukünftige Entwicklungen sollten daher, potenziell durch eine Verschlankung der Systeme, auf eine Reduktion der Investitions- und laufenden Kosten abzielen.

**Graphic abstract:**

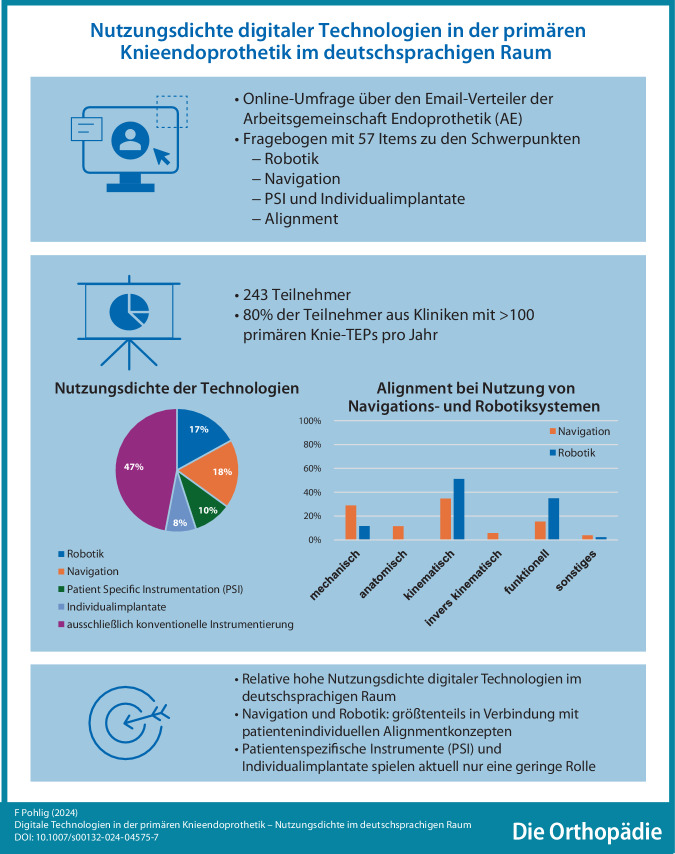

**Zusatzmaterial online:**

Zusätzliche Informationen (die komplette Umfrage) sind in der Online-Version dieses Artikels (10.1007/s00132-024-04575-7) enthalten.

## Abstract (en)

**Background:**

Digital tools are being increasingly used worldwide in primary knee arthroplasty. This study aimed to analyze the utilization density of digital tools, the preferred alignment strategies, and the obstacles and benefits of implementing these technologies in German-speaking countries.

**Materials and methods:**

An online survey with 57 questions about digital tools in primary knee arthroplasty and their usage was conducted among members of the Arthroplasty Working Group (AE). The survey included questions on navigation, robotics, patient-specific instruments, individualized implants, and augmented reality.

**Results:**

The survey revealed that 18% of hospitals use navigation and 17% use robotic systems in primary total knee arthroplasty surgery. The main reasons for not implementing supportive technologies were high acquisition and ongoing costs, as well as longer surgical duration. Patient-specific instruments and individualized implants currently play a minor role. Patient-specific alignment strategies, such as kinematic (navigation: 35%; robotics: 44%) and functional alignment (navigation: 15%; robotics: 35%), are preferred in this context. With conventional instrumentation predominantly mechanical alignment was applied (79%).

**Discussion:**

The results indicate a relatively high utilization density of digital tools, which are mainly used to perform personalized alignment strategies in primary knee arthroplasty in German-speaking countries. This was particularly evident in high-volume hospitals. Economic aspects were the main reasons for not using these technologies. Future developments should aim to simplify the systems and thus achieve improved cost efficiency.

## Hintergrund und Fragestellung

In den letzten Jahrzehnten hat sich die Knieendoprothetik deutlich weiterentwickelt, sowohl bezüglich der chirurgischen Technik als auch der verwendeten Implantate. Diese Entwicklungen haben vor allem zu einer erhöhten Lebensdauer der Prothesen geführt [3, 28]. Trotz dieser Erfolge konnte die Zufriedenheitsrate der Patienten nur geringfügig verbessert werden [6, 17, 30]. Somit verbleibt die Herausforderung der Optimierung der klinischen Ergebnisse. Ansatzpunkte könnten die Verbesserung der chirurgischen Präzision sowie die Rekonstruktion der individuellen Anatomie der einzelnen Patienten sein. In diesem Zusammenhang haben digitale Technologien, wie computergestützte Navigationssysteme und roboterassistierte Chirurgie, an Bedeutung gewonnen. Diese Technologien bieten vor allem das Potenzial, moderne individuelle Alignmentkonzepte präzise umzusetzen [8, 10, 36].

Die Implementierung digitaler Technologien in der orthopädischen Chirurgie variiert jedoch erheblich, beeinflusst durch eine Reihe von Faktoren wie Kosten, Zugänglichkeit der Technologie und nicht zuletzt durch die bisher eingeschränkte Evidenz für deren Wirksamkeit [1, 33, 34]. Im deutschsprachigen Raum bietet die starke medizinische Infrastruktur eine gute Basis für die Nutzung fortschrittlicher chirurgischer Hilfsmittel. Andererseits besteht ein hoher wirtschaftlicher Druck auf die Kliniken, wodurch technologische Innovationen zunehmend schwieriger zu implementieren sind. Bisher ist unklar, inwieweit digitale Technologien in der Praxis angenommen und angewendet werden, welche Alignmentphilosophien bevorzugt werden und welche spezifischen Herausforderungen die breitere Implementierung dieser Tools behindern [4, 19].

Ziel dieser Studie ist, den Einsatz digitaler Technologien in der primären Knieendoprothetik im deutschsprachigen Raum zu analysieren. Insbesondere sollen die Verbreitung von Robotik und Navigation, die vorherrschenden Alignmentstrategien und die Gründe für oder gegen die Nutzung dieser Technologien untersucht werden. Darüber hinaus werden die potenziellen Auswirkungen der digitalen Technologien auf die Präzision, Patientenzufriedenheit und Langzeitergebnisse diskutiert, um ein umfassendes Bild der Rolle digitaler Tools in der modernen Knieendoprothetik zu zeichnen.

## Studiendesign und Untersuchungsmethoden

### Studiendesign und Teilnehmer

Die vorliegende Studie basiert auf einer Querschnittsanalyse, die mittels einer Online-Umfrage durchgeführt wurde. Zielgruppe der Umfrage waren Orthopäden und Unfallchirurgen aus dem deutschsprachigen Raum (v. a. Deutschland, Österreich, Schweiz) die in Kliniken und Praxen tätig sind, welche primäre Knieendoprothetik anbieten. Die Einladung zur Teilnahme an der Umfrage erfolgte über den E‑Mail-Verteiler der Arbeitsgemeinschaft Endoprothetik (AE). Zur Vermeidung einer Überrepräsentanz einzelner Aspekte wurde in der Einladung darum gebeten, dass lediglich ein Fragebogen pro Endoprothetikzentrum eingereicht wird. Aufgrund der Anonymität der Umfrage war eine direkte Kontrolle diesbezüglich nicht möglich. Rückschlüsse auf die weitgehende Einhaltung dieser Aufforderung konnten jedoch durch den Vergleich der Ergebnisse mit öffentlich zugänglichen endoCert- und EPRD-Daten gezogen werden.

Die Studie wurde im Einklang mit den ethischen Standards der Deklaration von Helsinki durchgeführt. Da die Umfrage anonym war und keine sensiblen persönlichen Daten erfasst wurden, war kein Ethikvotum erforderlich. Die Teilnehmer wurden über den Zweck der Studie, die Freiwilligkeit der Teilnahme und den Umgang mit den gesammelten Daten informiert.

### Umfrageinstrument

Die Umfrage wurde mithilfe der Plattform SurveyMonkey® durchgeführt. Der Fragebogen enthielt insgesamt 57 Fragen zu verschiedenen Bereichen (Suppl. 1):

- Kennzahlen des Endoprothetikzentrums (z. B. Anzahl der jährlichen Knieprothesenimplantationen, Zertifizierungen)
- Nutzung digitaler Tools in der primären Knieendoprothetik, einschließlich Robotik, Navigation und Individualprothesen oder -schnittblöcke
- Präferierte Alignmentphilosophien mit den jeweiligen Tools
- Gründe für die Nutzung oder Ablehnung der Technologien
- Subjektiv wahrgenommene Vorteile und Herausforderungen der digitalen Technologien

Die Fragen wurden als Multiple-Choice, Skala-Bewertungen und offene Fragen gestaltet, um quantitative sowie qualitative Daten zu erfassen. Zu jedem digitalen Tool, beispielsweise Navigations- oder Robotiksystem, musste zunächst die Frage beantwortet werden, ob diese Technologie in der eigenen Klinik vorgehalten wird. Nur bei Vorhandensein der Technologie wurden weitere Fragen zu Nutzungsaspekten und Alignment angezeigt, sodass diese Aspekte nur von Nutzern der jeweiligen Technologien beantwortet werden konnten.

### Datenerhebung

Die Umfrage war über einen Zeitraum von 3 Wochen zugänglich. Um die Anonymität der Teilnehmer zu gewährleisten, wurden keine persönlich identifizierbaren Informationen gesammelt. Die Teilnahme war freiwillig, und es wurden keine Anreize für die Teilnahme angeboten. Die Datensammlung erfolgte automatisch über die Umfrageplattform, wobei die Einhaltung der Datenschutzgrundverordnung (DSGVO) sichergestellt wurde. Die erhobenen Datensätze können auf begründete Anfrage in anonymisierter Form beim korrespondierenden Autor angefordert werden. Die Daten befinden sich auf einem Datenspeicher der Klinik für Orthopädie und Sportorthopädie des Klinikums rechts der Isar der Technischen Universität München.

### Datenanalyse

Die gesammelten Daten wurden zunächst auf Vollständigkeit und Konsistenz geprüft. Quantitative Antworten wurden hauptsächlich mit deskriptiven Statistiken analysiert. Offene Antworten wurden mittels qualitativer Inhaltsanalyse ausgewertet, um Hauptthemen und Trends zu identifizieren. Die statistische Analyse wurde mit der Software SPSS (Version 25.0, IBM Corp., Armonk, NY, USA) durchgeführt.

## Ergebnisse

Insgesamt nahmen 243 Endoprothetiker an der Umfrage teil. Knapp 90 % der Befragten gab an, dass aus ihren Kliniken Daten an das EPRD übermittelt werden. 71 % der Kliniken waren zum Zeitpunkt der Umfrage als Endoprothetikzentrum (endoCert) zertifiziert. Die Ergebnisse zum Operationsvolumen der Kliniken der Umfrageteilnehmer sind in Tab. 1 zusammengefasst.

**Tab. 1:** Operationsvolumen der Kliniken der befragten Ärzte in der primären Knieendoprothetik pro Jahr

Anzahl der primären Knietotalendoprothesen pro Jahr (Klinik)	Anzahl Beantwortungen (*n*)	Prozentualer Anteil (%)
1–49	6	3
50–99	42	17
100–199	56	23
200–499	90	37
>499	49	20
*Gesamt*	243	100

**Abb. 1:**
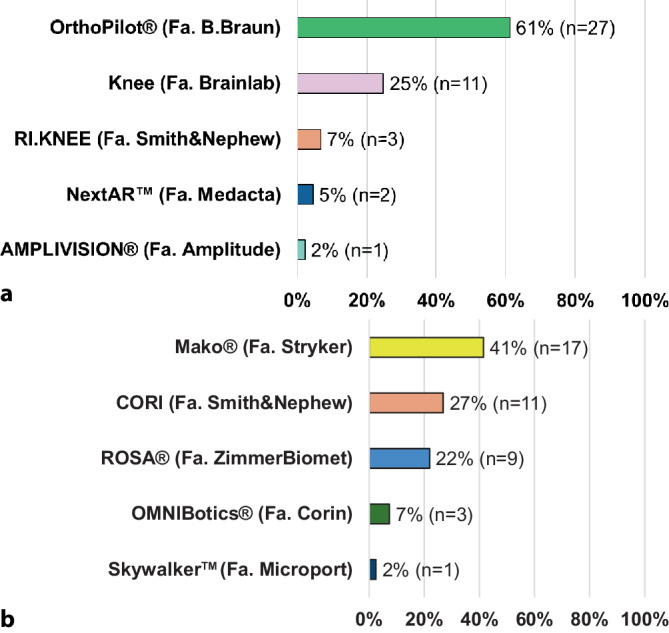
Prozentualer Anteil der Nutzung verschiedener (**a**) Navigations- und (**b**) Robotiksysteme (*n* = absolute Anzahl)

**Abb. 2:**
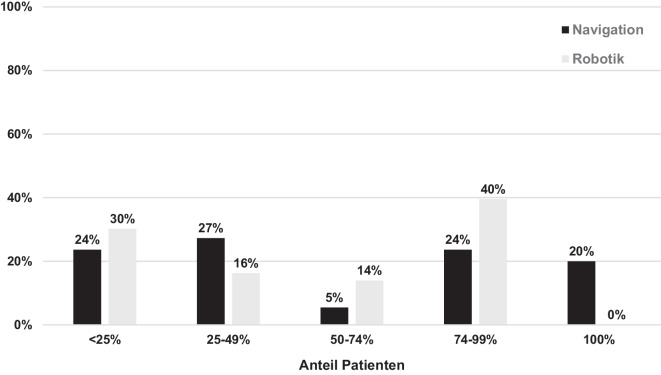
Anteil der Patienten, die in Kliniken mit vorhandener Hardware mit Unterstützung eines Navigations- oder Robotiksystems versorgt werden

Von den befragten Kliniken nutzen 18 % ein Navigationssystem für die primäre Knieendoprothetik. Die Nutzungsverteilung der verschiedenen Systeme ist in Abb. 1a dargestellt. In 17 % der Kliniken werden Robotiksysteme unterschiedlicher Hersteller eingesetzt. Die weiteste Verbreitung zeigte sich hier für die Systeme Mako® (Fa. Stryker, Kalamazoo, MI, USA), Cori (Smith&Nephew, London, UK) und Rosa® (Fa. Zimmer, Warsaw, IN, USA) (Abb. 1b). In den Kliniken mit Navigations- oder Robotik-Hardware erfolgt deren Einsatz zum Teil aber nur in begrenztem Maße (Abb. 2). Die Hauptgründe für die nur partielle Nutzung trotz der vorgehaltenen Technik waren der Zeitaufwand (Navigation: 29 %, Robotik: 40 %) und die zusätzlichen Kosten (Navigation: 11 %, Robotik: 21 %).

**Abb. 3:**
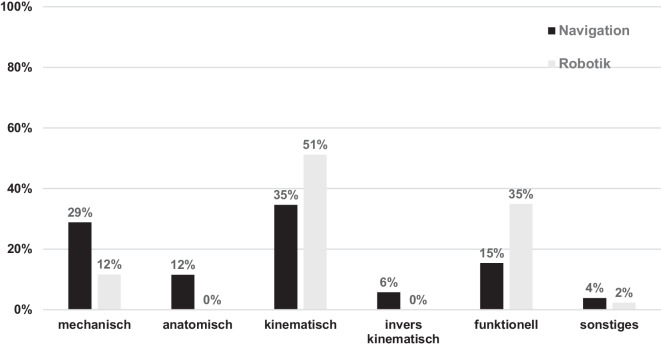
Bevorzugte Alignmentstrategien bei Anwendung von Navigations- und Robotiksystemen (kinematisches und invers kinematisches Alignment werden in einem Großteil der Kliniken mit Grenzen – „restricted“– eingesetzt)

In Zusammenhang mit Robotiksystemen werden von den Befragten patientenindividuelle Alignmentphilosophien wie kinematisches Alignment (51 %) oder funktionelles Alignment (35 %) präferiert (Abb. 3). Traditionell mechanische Ansätze spielen in der Robotik im deutschsprachigen Raum allenfalls eine untergeordnete Rolle (ca. 12 %). Zwar werden auch bei reiner Navigation häufig patientenindividuelle Ansätze verfolgt (kinematisches Alignment: 35 %, funktionelles Alignment 15 %), in knapp 30 % der Kliniken wird jedoch auch mit einem Navigationssystem ein mechanisches Alignment präferiert. Für beide Technologien, Navigation und Robotik, wurde im Zusammenhang mit kinematischem Alignment in über 70 % angegeben, dass Grenzen im Sinne eines „restricted kinematic alignment“ eingehalten werden. Für Robotiksysteme konnte eine 100 %ige Nutzung von „Fixed-bearing“-Implantaten ermittelt werden, für Navigationssysteme lag dieser Wert bei 69 %. Technologieübergreifend werden kreuzbanderhaltende Implantate („cruciate retaining“ = CR) bevorzugt (Navigation: 60 %, Robotik: 51 %). „Posterior-stabilized“ (PS) Implantate werden mit Robotiksystemen häufiger genutzt als mit Navigationssystemen (35 % vs. 15 %).

Der Anteil unikondylärer Prothesen an der Gesamtzahl der primären Knie-TEP lag bei einer großen Mehrheit der Kliniken unter 25 %. Lediglich 4 Kliniken gaben einen Anteil unikondylärer Prothesen zu bikondylären Versorgungen von über 50 % an. Auch werden nur in 14 % der Kliniken unikondyläre Schlittenprothesen mit einem Robotiksystem implantiert.

Unter den Umfrageteilnehmern, die keine Navigation oder Robotik, sondern ausschließlich eine manuelle Instrumentierung für die primäre Knie-TEP-Implantation nutzen, waren die hohen Anschaffungskosten, die laufenden Kosten, der zusätzliche Zeitaufwand sowie der derzeit noch fehlende Nachweis des medizinischen Nutzens die häufigsten Gründe, die genannten Technologien nicht zu implementieren. In der Bewertung, ob ein medizinischer Vorteil oder eher ein werbetechnischer Nutzen bestehen würde, zeigt sich eine relativ ausgewogene Meinung mit nur leichter Tendenz in Richtung Marketingtool. Deutlich ist hingegen, dass die konventionell operierenden Kliniken weiterhin überwiegend auf ein klassisches mechanisches Alignment, mit allenfalls leichten Adaptationen, setzen (79 %). Nur etwa 18 % streben mit konventioneller Instrumentierung ein kinematisches oder funktionelles Alignment an.

Patientenspezifische Instrumente („patient specific instrumentation“ = PSI) und Individualimplantate werden nur in wenigen Kliniken eingesetzt (PSI: 10 %, Individualimplantate: 5 %). Mittels PSI wird am häufigsten ein kinematisches Alignment verfolgt, mit Individualimplantaten wird vielfach ein mechanisches Alignment präferiert.

Augmented-Reality-Systeme kommen bisher in den Kliniken von lediglich 3 Befragten zum Einsatz (1 %). Als wichtigste Gründe für den Einsatz wurden Ausbildung, Forschung und Optimierung der operativen Genauigkeit genannt.

## Diskussion

Die Nutzung digitaler Technologien der Knieendoprothetik, wie sie in dieser Studie dokumentiert wurde, spiegelt einen globalen Trend zur Integration fortschrittlicher Technologien in die orthopädische Chirurgie wider. Im deutschsprachigen Raum konnte ein vergleichsweise hoher Grad der Implementierung dieser Technologien in der Knieendoprothetik gezeigt werden. Vor allem in Zusammenhang mit Robotik- und Navigationssystemen konnte in dieser Studie zudem ein deutlicher Trend zu patientenindividuellen Alignmentstrategien identifiziert werden im Vergleich zum weiterhin meist mechanischen Alignment bei konventioneller Instrumentierung.

Insgesamt wurden 243 Beantwortungen der Umfrage erfasst. Im Aufruf zur Teilnahme wurde explizit dazu aufgefordert, dass pro Klinik nur eine Beantwortung durchgeführt werden soll. Eine Überprüfung war aufgrund der anonymen Umfrage aber nicht möglich. Bei rund 500 Kliniken, die in Deutschland 2023 nach endoCert zertifiziert waren [11], wären 50 % dieser Kliniken in der Umfrage erfasst. Einschränkend ist hier aber zu erwähnen, dass nur 71 % der Befragten angegeben haben, dass ihre Klinik nach endoCert zertifiziert ist. 57 % der Umfrageteilnehmer gaben aber zudem an, dass an ihrer Klinik mehr als 200 primäre Knie-TEP pro Jahr implantiert werden. In der Zusammenschau kann somit von einer guten Repräsentativität der Ergebnisse ausgegangen werden.

In 17 % der Kliniken wird Robotik und in 18 % werden Navigationssysteme eingesetzt. Dies stellt einen deutlichen Wandel im Bereich der digitalen Assistenzsysteme dar. So konnten Schnurr et al. 2022 Navigationssysteme und Individualimplantate als wichtigste moderne Technologien in der primären Knieendoprothetik in Deutschland identifizieren [29]. Ein deutlicher Anstieg der Robotiksysteme wurde in dieser Umfragestudie jedoch bereits prognostiziert. Verglichen mit der Nutzungsdichte anderer Länder, scheint hierzulande eine relativ hohe Innovationsbereitschaft bezüglich Robotiksystemen vorzuliegen. Dies bestätigt sich in einer aktuellen Literaturanalyse von Kow et al. [18]. Hier zeigte sich, dass hinter den USA, Großbritannien und China die meisten Publikationen zum Thema roboterassistierte Endoprothetik von deutschen Arbeitsgruppen veröffentlicht wurden. Obwohl ein gewisser Trend abgeleitet werden kann, so stellt diese bibliographische Analyse sicher kein direktes Abbild der Versorgungsrealität dar. Australien wird in dieser Arbeit lediglich an fünfter Stelle aufgeführt, obwohl dort bereits 2022 über 30 % der jährlich etwa 800.000 primären Knie-TEPs mit Robotiksystemen durchgeführt wurden [2]. Die in dieser Studie ermittelte Nutzungsdichte von Robotik und Navigation wird zudem dadurch eingeschränkt, dass über 50 % der Befragten in Zentren mit relativ hohem Operationsvolumen tätig waren. Gerade in diesen Einrichtungen dürfte die Bereitschaft zur Investition in neue Technologien höher sein als an kleineren Kliniken mit niedrigem Operationsvolumen. Interessant ist, dass die vorgehaltenen Robotik- und Navigationssysteme in weniger als der Hälfte der Kliniken bei mehr als 75 % der behandelten Patienten eingesetzt werden. Somit scheint nicht nur die Anfangsinvestition in diese Technologien eine Barriere darzustellen [23]. Die aktuelle Studie hebt hervor, dass auch laufende Kosten und zusätzlicher Zeitaufwand wichtige Barrieren für die breitere Adoption in der täglichen Praxis darstellen. Diese Beobachtung steht im Einklang mit der Literatur, die aufzeigt, dass trotz der potenziellen Vorteile die Kosten und die Lernkurve für die Anwendung dieser Technologien signifikante Herausforderungen darstellen [7, 23, 26]. Zukünftige Entwicklungen sollten daher auf eine Verschlankung der Systeme zur Reduktion der Anfangsinvestition aber auch der laufenden Kosten und des intraoperativen Zeitaufwands abzielen. Nur so kann langfristig eine breite Anwendung aber auch Kosteneffektivität im Kontext des Gesundheitssystems erzielt werden.

Interessanterweise konnten deutliche Unterschiede in der Häufigkeitsverteilung verschiedener Alignmentphilosophien bei Anwendung von Navigations- im Vergleich zu Robotiksystemen gezeigt werden. So wurde bei etwa 30 % der Patienten mittels Navigation ein mechanisches Alignment umgesetzt. Dies entspricht eher der klassischen Philosophie, dass durch die Navigation beim mechanischen Alignment signifikante Abweichungen der Implantatposition und der Beinachse im Vergleich zur manuellen Instrumentierung vermieden werden sollen [21]. Im Gegensatz dazu wurde das mechanische Alignment in Zusammenhang mit Robotik kaum genannt. Hier finden, analog zur aktuellen Literatur, größtenteils patientenspezifische Alignmentkonzepte Anwendung [22, 25]. Hauptsächlich wurden in Zusammenhang mit Robotiksystemen kinematisches und funktionelles Alignment genannt. Hervorzuheben ist, dass keine Klinik mit Robotiksystem ein invers kinematisches Alignment anzuwenden scheint [35]. Dies kann, zumindest zum Teil, dadurch erklärt werden, dass verschiedene Autoren ihr Konzept des funktionellen Alignments mehr auf Basis eines invers kinematischem Alignments beschreiben [22, 27, 32]. Somit kann in dieser Studie von einer gewissen Vermischung des invers kinematischen und des funktionellen Alignments ausgegangen werden. Interessant ist aber auch der hohe Anteil des kinematischen Alignments. Gemäß der von Howell et al. beschriebenen Prinzipien würde dies bedeuten, dass die präarthrotische knöcherne Konfiguration ohne Grenzen rekonstruiert werden soll [13, 14]. Eine potenzielle ligamentäre Imbalance durch eine über Jahre bestehende Gonarthrose mit progredienter Achsdeviation findet bei diesem Konzept allenfalls geringe Beachtung. In der vorliegenden Studie gab jedoch eine überwiegende Mehrheit an, dass hinsichtlich der Implantatposition und des Alignments Limits eingehalten werden. Zudem ermöglichen die meisten Robotiksysteme eine intraoperative Erfassung der Bandspannung. Es ist davon auszugehen, dass bei einem Teil der Patienten eine Balancierung der Bandspannung ausschließlich über die Veränderung der tibialen Resektionsebene unter gleichzeitiger Einhaltung von Limits nicht möglich ist. Somit ist auch diesbezüglich bei einer Mehrzahl der Anwender von einer Form des funktionellen als eines strikt kinematischen Ansatzes auszugehen. Dies spiegelt aber auch die Schwierigkeit der absoluten Kategorisierung der unterschiedlichen patientenspezifischen Alignmentkonzepte wider, da die Grenzen vielfach fließend verlaufen und eine klare Einordnung schwierig sein kann [31].

Die Ergebnisse hinsichtlich der Implantatwahl sind wenig überraschend. Bei den meisten Robotikeinheiten handelt es sich um geschlossene Systeme, sodass lediglich die vom Hersteller angebotenen Implantatmodelle angewendet werden können [15]. Der hohe Anteil von Implantaten mit fixierter Plattform ist somit mehr system- als operateursspezifisch zu werten. Bei offenen Systemen oder dem Angebot beider Plattformen innerhalb eines Systems wäre möglicherweise von einem höheren Nutzungsanteil von Implantaten mit mobiler Plattform auszugehen. Allerdings ist ein gewisser Widerspruch zwischen einem patientenindividuellen Alignment mit möglichst physiologischer Rekonstruktion der Biomechanik und dem Konzept einer mobilen Plattform anzumerken. Aktuelle Metaanalysen zeigen keinen eindeutigen Vorteil zugunsten von Implantaten mit fixierter oder mobiler Plattform. Jedoch beziehen sich diese Ergebnisse vornehmlich auf mechanisches Alignment und können damit nicht uneingeschränkt auf patientenindividuelle Alignmentphilosophien übertragen werden [12, 24].

Bei Anwendung von Individualimplantaten wurde in der aktuellen Umfrage großteils ein mechanisches Alignment präferiert. Allerdings ist dieses Ergebnis im Kontext der Marktsituation zu interpretieren. Zum Zeitpunkt der Umfrage waren zwei Individualimplantatsysteme in Deutschland verfügbar: iTotal® (Fa. Conformis, Bedford, MA, USA) und ORIGIN® (Fa. Symbios, Yverdon-les-Bains, Schweiz). Conformis war mit dem iTotal®-System lange Zeit Marktführer im deutschsprachigen Raum. Mit diesem System wurde ein mechanisches beziehungsweise durch das geteilte Inlay ein anatomisches Alignmentkonzept verfolgt, was die Ergebnisse der Umfrage bestätigt. Allerdings hat sich Conformis inzwischen vom deutschen Markt zurückgezogen, sodass nur noch das ORIGIN®-Implantatsystem der Firma Symbios mit patientenindividuellem Alignmentkonzept in Deutschland verfügbar ist. Eine zukünftige Verschiebung zu ausschließlich individuellem Alignment bei Anwendung von Individualimplantaten ist somit nicht nur der Präferenz der Anwender, sondern auch der Marktsituation geschuldet. Außerhalb des deutschsprachigen Raums sind meist beide Systeme verfügbar, sodass weltweit weiterhin sowohl mechanisches als auch patientenindividuelles Alignment mit Individualimplantaten angewandt werden wird.

Interessanterweise wurde für die Einführung von Augmented Reality in einer erst 2022 publizierten Umfragestudie von Schnurr und Kollegen eine ähnlich starke Entwicklung wie für die Robotik prognostiziert [29]. Dies ließ sich mit den aktuellen Daten nicht bestätigen. Die wenigen Anwender von Augmented-Reality-Systemen nannten Ausbildung als einen der wichtigsten Gründe für die Implementierung. Aber auch Robotik- und Navigationssysteme sind für die Ausbildung junger Kollegen sehr geeignet, da hier in Echtzeit die Auswirkung auch kleiner Veränderungen der Implantatposition auf Alignment, Spaltweiten und Bandspannung erlernt werden können. Kritisch ist aber eine übertrieben auf Assistenzsysteme fokussierte Ausbildung zu betrachten, da die konventionelle Implantationstechnik möglicherweise nicht mehr ausreichend vermittelt wird. Dies bestätigt sich in einer aktuellen Studie von Duensing et al., in der 25 % der befragten Assistenzärzte eine zu geringe Ausbildung der konventionellen Technik beklagen [9].

Die abschließende Frage zur Einschätzung von medizinischem Nutzen oder reinem Marketing von Robotik in der Knieendoprothetik wurde relativ ausgeglichen beantwortet. Dies steht im Einklang mit einer von Sherman und Wu publizierten Umfrage unter Mitgliedern der American Association of Hip and Knee Surgeons (AAHKS), in der ebenfalls etwa 50 % der Befragten die Robotik vorrangig als Marketingtool einschätzten [9, 33]. Bestätigt wird diese Einschätzung durch aktuelle Metaanalysen, in denen zwar eine erhöhte Präzision, aber kein signifikant verbessertes klinisches Outcome nachgewiesen werden konnte [1, 34, 36]. Einzelstudien aber konnten durchaus auch klinische Vorteile der roboterassistierten Knieendoprothetik demonstrieren [5, 16, 20]. Diese Diskrepanz ist, nach Ansicht der Autoren, unter anderem auf die großen Unterschiede zwischen den aktuell angewandten Alignmentstrategien zurückzuführen. Die Identifikation eindeutiger Vorteile, vor allem in systematischen Reviews und Metaanalysen, ist somit schwierig. Zudem wird das eigene Konzept von den meisten Operateuren mutmaßlich bei allen Patienten angewandt, obwohl weiterhin unklar ist, ob jede Alignmentstrategie und jedes Implantat für jeden Patienten gleichermaßen geeignet ist.

Für die vorliegende Studie muss auf einige Limitationen hingewiesen werden. Erstens zeigen die hohen Operationsvolumina, die von einer Mehrzahl der Befragten angegeben wurden, dass die dargestellte Verbreitung moderner Technologien hauptsächlich für Zentren gilt. Es ist davon auszugehen, dass die Implementierung digitaler Assistenzsysteme in der primären Knieendoprothetik in kleineren Kliniken nicht in gleichem Maße angekommen ist. Zweitens wurden vorgehaltene Assistenzsysteme in den meisten Kliniken nicht bei allen Patienten eingesetzt. Damit ist der prozentuale Anteil der Versorgungen geringer als bei reiner Betrachtung der Verbreitung der Hardware. Drittens wurden zum Alignment lediglich die bekannten Philosophien abgefragt. Eine Erörterung der persönlichen Umsetzung der Alignmentstrategie durch die Befragten ist nicht erfolgt. Somit kann eine fehlerhafte Zuordnung der angewandten Alignmentphilosophie und damit eine potenzielle Überrepräsentanz einzelner Strategien nicht ausgeschlossen werden.

## Schlussfolgerung

- Vergleichsweise hohe Nutzungsdichte digitaler Technologien in der Knieendoprothetik im deutschsprachigen Raum.
- Navigation und Robotik werden am häufigsten genutzt und hiermit größtenteils patientenindividuelle Alignmentkonzepte umgesetzt.
- Patientenspezifische Instrumente und Implantate spielen aktuell nur eine untergeordnete Rolle.
- Augmented-Reality-Anwendungen werden bislang kaum eingesetzt.

## Supplementary Information

Suppl. 1 Fragebogen mit 57 Items

## Abkürzungen

AAHKS: American Association of Hip and Knee Surgeons
AE: Arbeitsgemeinschaft Endoprothetik
CR: Cruciate retaining
EPRD: Endoprothesenregister Deutschland
Knie-TEP: Knietotalendoprothese
PS: Posterior stabilized
PSI: Patient specific instrumentation
