# Supplementary material for: Digital tools in primary total knee arthroplasty—Prevalence in the German-speaking region
Source: Orthopadie (Heidelb). 2024 Nov 1;53(12):935–44. [Article in German] doi: 10.1007/s00132-024-04575-7 (PMC11604827; doi:10.1007/s00132-024-04575-7)
Supplement: Supplementary file 1 — Suppl. 1 Fragebogen mit 57 Items [file 132_2024_4575_MOESM1_ESM.pdf]

Allgemeine Informationen

* 1. Wieviele primäre Knieprothesenimplantationen pro Jahr führen Sie an Ihrer Klinik durch?

- 1-49
- 50-99
- 100-199
- 200-499
- >500

* 2. Geben Sie die Daten in das ERPD ein?

- Ja
- Nein

* 3. Ist Ihre Klinik EndoCert zertifiziert?

- Ja
- Nein

Frage Navigation

* 4. Nutzen Sie an Ihrer Klinik für die Knieendoprothetik regelmäßig ein Navigationssystem (ohne Robotik)?

- ◯ Ja
- ◯ Nein

Navigation

5. Bei welchem Prozentsatz der an Ihrer Klinik implantierten Knieprothesen wird ein Navigationssystem genutzt?

- ○ < 25%
- ○ 25-49%
- ○ 50-74%
- ○ 75-99%
- ○ 100%

6. Welches Navigationssystem nutzen Sie?

7. Bei welchen Voraussetzungen wird an Ihrer Klinik in der Regel KEINE Navigation genutzt bzw. welches sind Gründe, weshalb nicht bei 100% eine Navigation genutzt wird? (Mehrfachauswahl möglich)

- ☐ Ausgeprägte Varusgonarthrose
- ☐ Ausgeprägte Valgusgonarthrose
- ☐ posttraumatische Gonarthrose
- ☐ Kosten
- ☐ Zeitaufwand
- ☐ Erweiterte Bildgebung präoperativ
- ☐ Allergie
- ☐ Sonstiges (bitte angeben)

8. Bei welchen Fällen wird an Ihrer Klinik vorzugsweise ein Navigationssystem genutzt? (Mehrfachauswahl möglich)

- ☐ Ausgeprägte Varusgonarthrose
- ☐ Ausgeprägte Valgusgonarthrose
- ☐ posttraumatische Gonarthrose
- ☐ Privatpatienten
- ☐ Sonstiges (bitte angeben)

9. Welche Ausrichtungsphilosophie nutzen Sie an Ihrer Klinik vornehmlich in Verbindung mit der Navigation?

- ◯ mechanisch Femurrotation bandreferenziert,
- ◯ mechanisch Femurrotation knöchern referenziert
- ◯ anatomisch
- ◯ kinematisch
- ◯ reverse kinematisch
- ◯ funktionell
- ◯ Sonstiges (bitte angeben)

10. Verfolgen Sie die oben angegebene Alignmentphilosophie mit Limits (z.B. restricted kinematisch)?

- ◯ Ja
- ◯ Nein
- ◯ Nicht zutreffend (bei z.B. mechanischem Alignment)

11. Welche Art von primärem Kniesystem nutzen Sie hauptsächlich?

- ◯ Fixed Bearing
- ◯ Mobile Bearing

12. Was nutzen Sie hauptsächlich?

- ◯ Cruciate Retaining
- ◯ Posterior Stabilized
- ◯ Ultrakongruent
- ◯ Medial Pivot

Frage Robotik

* 13. Nutzen Sie an Ihrer Klinik für die Knieendoprothetik regelmäßig ein Robotiksystem?

- ◯ Ja
- ◯ Nein

Robotik

14. Bei welchem Prozentsatz der an Ihrer Klinik implantierten Knieprothesen wird ein Robotiksystem genutzt?

- ○ < 25%
- ○ 25-49%
- ○ 50-74%
- ○ 75-99%
- ○ 100%

15. Welches Robotiksystem nutzen Sie?

[ ]

16. Bei welchen Voraussetzungen wird an Ihrer Klinik in der Regel KEINE Robotik genutzt bzw. welches sind Gründe, weshalb nicht bei 100% ein Robotiksystem genutzt wird? (Mehrfachauswahl möglich)

- ☐ Ausgeprägte Varusgonarthrose
- ☐ Ausgeprägte Valgusgonarthrose
- ☐ posttraumatische Gonarthrose
- ☐ Kosten
- ☐ Zeitaufwand
- ☐ Erweiterte Bildgebung präoperativ
- ☐ Allergie
- ☐ Sonstiges (bitte angeben)

[ ]

17. Wann nutzen Sie an Ihrer Klinik vorzugsweise ein Robotiksystem? (Mehrfachauswahl möglich)

- ☐ Ausgeprägte Varusgonarthrose
- ☐ Ausgeprägte Valgusgonarthrose
- ☐ posttraumatische Gonarthrose
- ☐ Privatpatienten
- ☐ Sonstiges (bitte angeben)

[ ]

18. Welche Ausrichtungsphilosophie nutzen Sie an Ihrer Klinik vornehmlich in Kombination mit Robotik?

- ◯ mechanisch Femurrotation bandreferenziert
- ◯ mechanisch Femurrotation knöchern referenziert
- ◯ adaptiert mechanisch
- ◯ anatomisch
- ◯ kinematisch
- ◯ reverse kinematisch
- ◯ funktionell
- ◯ Sonstiges (bitte angeben)

19. Verfolgen Sie die oben angegebene Alignmentphilosophie mit Limits (z.B. restricted kinematisch)?

- ◯ Ja
- ◯ Nein
- ◯ Nicht zutreffend (bei z.B. mechanischem Alignment)

20. Welche Art von primärem Kniesystem nutzen Sie hauptsächlich?

- ◯ Fixed Bearing
- ◯ Mobile Bearing

21. Was nutzen Sie hauptsächlich?

- ◯ Cruciate Retaining
- ◯ Posterior Stabilized
- ◯ Ultrakongruent
- ◯ Medial Pivot

Frage Schnittblöcke

* 22. Nutzen Sie an Ihrer Klinik für die Knieendoprothetik regelmäßig patientenspezifische Schnittblöcke?

◯ Ja

◯ Nein

Patientenspezifische Schnittblöcke

23. Bei welchem Prozentsatz der an Ihrer Klinik implantierten Knieprothesen nutzen Sie patientenspezifische Schnittblöcke?

- ◯ < 25%
- ◯ 25-49%
- ◯ 50-74%
- ◯ 75-99
- ◯ 100%

24. Welches System nutzen Sie?

[ ]

25. Bei welchen Voraussetzungen nutzen Sie an Ihrer Klinik in der Regel KEINE Individualschnittblöcke (Mehrfachauswahl möglich) bzw. welches sind Gründe, weshalb Sie nicht bei 100% Individualschnittblöcke anwenden?

- ☐ Ausgeprägte Varusgonarthrose
- ☐ Ausgeprägte Valgusgonarthrose
- ☐ posttraumatische Gonarthrose
- ☐ Kosten
- ☐ Zeitaufwand
- ☐ Erweiterte Bildgebung präoperativ
- ☐ Allergie
- ☐ Sonstiges (bitte angeben)

[ ]

26. Wann nutzen Sie an Ihrer Klinik vorzugsweise Individualschnittblöcke (Mehrfachauswahl möglich)?

- ☐ Ausgeprägte Varusgonarthrose
- ☐ Ausgeprägte Valgusgonarthrose
- ☐ posttraumatische Gonarthrose
- ☐ Privatpatienten
- ☐ Sonstiges (bitte angeben)

[ ]

27. Welche Ausrichtungsphilosophie nutzen Sie an Ihrer Klinik vornehmlich in Verbindung mit individuellen Schnittblöcken?

- ◯ mechanisch Femurrotation bandreferenziert,
- ◯ mechanisch Femurrotation knöchern referenziert
- ◯ adaptiert mechanisch
- ◯ anatomisch
- ◯ kinematisch
- ◯ reverse kinematisch
- ◯ funktionell
- ◯ Sonstiges (bitte angeben)

28. Verfolgen Sie die oben angegebene Alignmentphilosophie mit Limits (z.B. restricted kinematisch)?

- ◯ Ja
- ◯ Nein
- ◯ Nicht zutreffend (bei z.B. mechanischem Alignment)

29. Welche Art von primärem Kniesystem nutzen Sie hauptsächlich?

- ◯ Fixed Bearing
- ◯ Mobile Bearing

30. Was nutzen Sie hauptsächlich?

- ◯ Cruciate Retaining
- ◯ Posterior Stabilized
- ◯ Ultrakongruent
- ◯ Medial Pivot

Frage Individualimplantate

* 31. Nutzen Sie an Ihrer Klink für die Knieendoprothetik regelmäßig Individualimplantate?

◯ Ja

◯ Nein

Patientenspezifische Implantate

32. Bei welchem Prozentsatz der an Ihrer Klinik implantierten Knieprothesen nutzen Sie patientenspezifische Implantate?

- ◯ < 25%
- ◯ 25-49%
- ◯ 50-74%
- ◯ 75-99
- ◯ 100%

33. Welches System nutzen Sie?

[ ]

34. Bei welchen Voraussetzungen nutzen Sie an Ihrer Klinik in der Regel KEINE Individualimplantate (Mehrfachauswahl möglich) bzw. welches sind Gründe, weshalb Sie nicht bei 100% Individualimplantate anwenden?

- ☐ Ausgeprägte Varusgonarthrose
- ☐ Ausgeprägte Valgusgonarthrose
- ☐ posttraumatische Gonarthrose
- ☐ Kosten
- ☐ Zeitaufwand
- ☐ Erweiterte Bildgebung präoperativ
- ☐ Allergie
- ☐ Sonstiges (bitte angeben)

[ ]

35. Wann nutzen Sie an Ihrer Klinik vorzugsweise Individualimplantate (Mehrfachauswahl möglich)?

- ☐ Ausgeprägte Varusgonarthrose
- ☐ Ausgeprägte Valgusgonarthrose
- ☐ posttraumatische Gonarthrose
- ☐ Privatpatienten
- ☐ Sonstiges (bitte angeben)

[ ]

36. Welche Ausrichtungsphilosophie nutzen Sie an Ihrer Klinik vornehmlich in Verbindung mit Individualimplantaten?

- ◯ mechanisch Femurrotation bandreferenziert,
- ◯ mechanisch Femurrotation knöchern referenziert
- ◯ adaptiert mechanisch
- ◯ anatomisch
- ◯ kinematisch
- ◯ reverse kinematisch
- ◯ funktionell
- ◯ Sonstiges (bitte angeben)

37. Verfolgen Sie die oben angegebene Alignmentphilosophie mit Limits (z.B. restricted kinematisch)?

- ◯ Ja
- ◯ Nein
- ◯ Nicht zutreffend (bei z.B. mechanischem Alignment)

38. Welche Art von primärem Kniesystem nutzen Sie hauptsächlich?

- ◯ Fixed Bearing
- ◯ Mobile Bearing

39. Was nutzen Sie hauptsächlich?

- ◯ Cruciate Retaining
- ◯ Posterior Stabilized
- ◯ Ultrakongruent
- ◯ Medial Pivot

Frage konventionelle Knieendoprothetik

* 40. Nutzen Sie an Ihrer Klinik für die Knieendoprothetik in der Regel keine digitalen Tools sondern hauptsächlich eine konventionelle Instrumentierung? **(Bitte nur mit "Ja" beantworten, wenn wirklich nahezu alle Knie-TEPs in konventioneller Technik implantiert werden.)**

- ◯ Ja
- ◯ Nein

Konventionelle Knieendoprothetik

41. Aus welchen Gründen nutzen Sie an Ihrer Klinik keine digitalen Tools in der Knieendoprothetik (Mehrfachauswahl möglich)?

- [ ] Hohe Anschaffungskosten Hardware
- [ ] Laufende Kosten
- [ ] Zeitaufwand
- [ ] Erweiterte Bildgebung präoperativ
- [ ] Kein eindeutiger medizinischer Nutzen
- [ ] Allergie
- [ ] Sonstiges (bitte angeben)

42. Welche Ausrichtungsphilosophie nutzen Sie an Ihrer Klinik vornehmlich in Verbindung mit konventioneller Instrumentierung?

- ○ mechanisch Femurrotation bandreferenziert,
- ○ mechanisch Femurrotation knöchern referenziert
- ○ adaptiert mechanisch
- ○ anatomisch
- ○ kinematisch
- ○ reverse kinematisch
- ○ funktionell
- ○ Sonstiges (bitte angeben)

43. Welches Implantatsystem verwenden Sie?

44. Welche Art von primärem Kniesystem nutzen Sie hauptsächlich?

- ○ Fixed Bearing
- ○ Mobile Bearing

45. Was nutzen Sie hauptsächlich?

- ○ Cruciate Retaining
- ○ Posterior Stabilized
- ○ Ultrakongruent
- ○ Medial Pivot

Frage unikondyläre Schlitten

* 46. Implantieren Sie an Ihrer Klinik auch unikondyläre Schlittenprothesen?

- ◯ Ja
- ◯ Nein

Unikondyläre Schlittenprothesen

47. Welchen prozentualen Anteil haben unikondyläre Schlittenprothesen an der gesamten primären Knieendoprothetik in Ihrer Klinik?

- ◯ <25%
- ◯ 25-49%
- ◯ 50-74%
- ◯ 75-99%
- ◯ 100%

48. Bei welchem Prozentsatz der an Ihrer Klinik implantierten unikondylären Schlittenprothesen nutzen Sie ein Robotiksystem?

- ◯ Es wird kein Robotik-System für unikondyläre Schlitten genutzt
- ◯ < 25%
- ◯ 25-49%
- ◯ 50-74%
- ◯ 75-99%
- ◯ 100%

49. Welches Robotiksystem nutzen Sie?

[ ]

50. Bei welchen Voraussetzungen nutzen Sie an Ihrer Klinik in der Regel KEINE Robotik (Mehrfachauswahl möglich) bzw. welches sind Gründe, weshalb Sie nicht bei 100% Robotik anwenden?

- ☐ Es wird kein Robotik-System für unikondyläre Schlitten genutzt
- ☐ Kosten
- ☐ Zeitaufwand
- ☐ Erweiterte Bildgebung präoperativ
- ☐ Metall-Allergie
- ☐ Sonstiges (bitte angeben)

[ ]

51. Welche Art von unikondylärem Kniesystem nutzen Sie an Ihrer Klinik hauptsächlich in Kombination <u>mit Robotik</u>?

- ◯ Es wird kein Robotik-System für unikondyläre Schlitten genutzt
- ◯ Fixed Bearing
- ◯ Mobile Bearing

52. Welche Art von unikondylärem Kniesystem nutzen Sie an Ihrer Klinik hauptsächlich bei konventioneller Technik?

- ◯ Fixed Bearing
- ◯ Mobile Bearing

53. Wie viele laterale unikondyläre Schlittenprothesen werden an Ihrer Klinik implantiert (prozentualer Anteil an der Gesamtzahl der unikondylären Schlitten)?

- ◯ 0-10%
- ◯ 11-25%
- ◯ 26-50%
- ◯ >50%

Augmented Reality

* 54. Nutzen Sie an Ihrer Klinik regelmäßig ein Augmented Reality System?

- ◯ Ja
- ◯ Nein

55. Welches Augmented Reality System nutzen Sie?

56. Wozu nutzen Sie ein Augmented Reality System?

- [ ] Aus- und Weiterbildung
- [ ] Optimierung der Genauigkeit bzw. Implantatlage
- [ ] Forschung
- [ ] Virtuelle Hospitationen
- [ ] Sonstiges (bitte angeben)

Abschluss

* 57. Wie beurteilen Sie die digitalen Tools (v.a. die Robotik) in Hinblick auf medizinischen Nutzen vs. Marketing?

100% Medizinischer Nutzen — 100% Marketing Tool
